# Bronchoscopy Without Sedation in Healthy Volunteers: A Viable Approach for Research

**DOI:** 10.1016/j.chpulm.2024.100075

**Published:** 2024-07-04

**Authors:** Dean Kellogg, Diego Maselli, Kevin Proud, Eusondia Arnett, Larry S. Schlesinger, Jay I. Peters

**Affiliations:** a University of Texas Health at San Antonio, San Antonio, TX; b South Texas Veterans Health Care System, San Antonio, TX; c Texas Biomedical Research Institute, San Antonio, TX

To the Editor:

Diagnostic bronchoscopy is a cornerstone of pulmonary medicine, but can induce anxiety for patients. Patient discomfort and coughing can lead to suboptimal bronchoscope wedging and can compromise diagnostic yield. The British Thoracic Society (grade B)^1^ and the American College of Chest Physicians^2^ recommend titration of IV moderate sedation to comfort, but this carries a range of potential adverse events, including respiratory depression, hypotension, and vomiting. In contrast, topical anesthesia offers a shorter duration of action and a wider therapeutic index.^3^ Although upper airway endoscopy is performed commonly without sedation, the tolerability of sampling the lower airways without sedation has not been studied systematically. Based on prior small studies of unsedated bronchoscopy in healthy volunteers,^4^ we designed this prospective study to optimize airway topical anesthesia using lidocaine solution delivered via a high-efficiency nebulizer, atomizer, and bronchoscope as well as jelly via lollypop and bronchoscope. Our aim was to describe the protocol for unsedated research bronchoscopy using solely topical lidocaine and to report the safety and efficacy of BAL in collecting human alveolar macrophages (HAMs).

## Methods

### Safety and Tolerability Assessment Before Study

Before conducting the study, unsedated bronchoscopy was performed on one investigator (J. I. P.; age, 68 years) to assess safety. The investigator reported a discomfort level of “1 to 2 out of 5,” and serum lidocaine was 2.2 μg/mL at the end of the procedure and undetectable 30 min later (toxic serum level, > 5 μg/mL). During protocol optimization before the study, we found that 4% nebulized lidocaine provided better anesthesia to the lower airways than 2% solution.

### Study Protocol

Eligible volunteers were between 18 and 50 years of age without medical comorbidities and in good health. All participants underwent a medical history interview, physical examination, serum chemistry analysis, spirometry, and exhaled CO_2_ testing.

**Figure 1:**
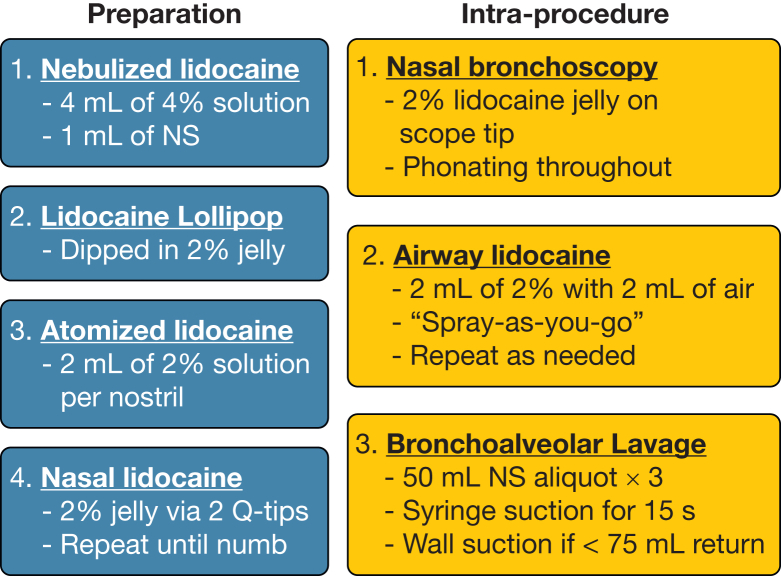
Diagram showing the topical anesthesia protocol. A nebulized solution made with 1 mL NS and 4 mL 4% lidocaine and delivered with a Monaghan Medical Aero Eclipse breath-actuated nebulizer with compressed air at 50 pounds per square inch and 8 L/min. A lollipop dipped in lidocaine was advanced repeatedly to the epiglottis until numb, and then 2 mL 2% lidocaine solution was atomized into both nostrils with a syringe and canonical device. Two lidocaine-coated sterile cotton swabs were inserted into each nostril for 1 to 2 min. Manual suction applied with a 60-mL syringe immediately after instillation. BAL samples were stored on ice before cell counting. The bronchoscope tip was coated with 2% lidocaine jelly. Airway lidocaine solution was instilled in six 2-mL aliquots of 2% lidocaine solution over the vocal cords, as well as the main and right middle carinas with a maximum of 10 to 15 sprays. After wedging in the right middle lobe, three 50-mL aliquots of sterile 0.9% NS were instilled rapidly over 5 to 10 s and suctioned manually after a 5-s dwell time using a 60-mL syringe. If the return was < 75 mL, wall suction was applied with a 40-mL lavage trap (occurred in one participant). NS = normal saline.

Bronchoscopy was conducted at the Barter Clinical Research Unit (South Texas Veterans Health Care System and University of Texas Health at San Antonio) under the supervision of the University of Texas Health at San Antonio Institutional Review Board (Identifier: HSC20170667H). Total lidocaine dose was 2.65 mg/kg (minimum toxic dose, 6.4 mg/kg). Topical anesthesia was achieved through sequential nebulized, oral, atomized, and bronchoscopic administration of lidocaine (Fig 1). Bronchoscopy was performed by an experienced pulmonologist using the Olympus BF-P60 bronchoscope (diameter, 4.9 mm) with a nasal approach. BAL was performed in the right middle lobe.

After bronchoscopy, the participants were observed for 2 h and did not eat until numbness had resolved. Telephone follow-up was conducted the following day to assess complications and discomfort level (1 = no discomfort, 5 = very uncomfortable).

BAL samples were transported on ice, centrifuged, and washed twice at 4 °C within 6 h, and the pellet was resuspended in Roswell Park Memorial Institute 1640 with penicillin G (10,000 U/mL). BAL cells were counted on a hemocytometer and 5 × 10^4^ cells were transferred to cytoslides. The cytoslides were dried, stained using HEMA 3 (Fisher Health Care), washed with water, dried, and examined with an AE2000 microscope (Motic).

## Results

The study volunteers (n = 25) were young (mean age, 27.4 years; range, 18-36 years) with equal sex distribution (48% female) and normal spirometry values: FEV_1_, 98% (range, 76%-115%); FEV_1_ to FVC ratio, 0.98 (range, 0.89-1.10). No participants who actively smoked were included as confirmed by exhaled carbon monoxide of < 5 parts per million. One patient with a history of anxiety withdrew from the study before evaluation. Details about the comfort, complications, and efficacy of bronchoscopy are provided in Table 1. Participants mostly reported mild discomfort (mean Likert value, 2.3), predominately from the passage through the turbinates. No immediate postprocedural complications occurred, nor did patients report any concerns on the following day. Mean room air oxygen saturation remained > 95% throughout. Two participants experienced transient desaturation to 88% to 89% oxygen saturation and recovered spontaneously without supplemental oxygen. One participant who reported 4 of 10 discomfort also experienced desaturation to 87% when coughing after the procedure. No changes from baseline were noted for BP or heart rate before or after the procedure.

**Table 1:** Bronchoscopy With Topical Anesthesia

Details	Data
Duration, min	7 (4-10)
Discomfort^a^	2.3 (1-4)
Oxygenation	
Desaturation < 89%	2 (8)
Lowest SpO_2_, %	96 (87-100)
Discharge SpO_2_, %	97 (88-100)
Procedure details	
Bronchoscopic lidocaine, mL	8 (6-12)
BAL return, mL	84 (45-110)
BAL return, % instilled	56 (30-73)
HAM yield, 10^6^ cells/mL	4.5 (1.2-8.6)
Cytospin confirmation, %^b^	89 (75-98)
Mucoid BAL return	5 (20)
Blood-tinged return^c^	1 (6)

Data are presented as No. (%) or mean (range). SpO_2_ = oxygen saturation; HAM = human alveolar macrophage.

a 1 = no discomfort to 5 = very uncomfortable.

b n = 20.

c Contaminated from nasal turbinate trauma.

Although 26% of BAL samples exhibited blood-mixed fluid or mucus, this did not have a detrimental impact on the yield of HAMs, which was satisfactory (mean, 4.5 × 10^6^ cells/mL). Cytospin analysis revealed that the mean percentage of macrophages in the BAL samples was 89% (range, 75%-98%).

## Discussion

In this study involving healthy volunteers, bronchoscopy without sedation or supplemental oxygen using multimodal topical lidocaine was well tolerated and proved to be a viable method for isolating HAMs. No procedures were terminated prematurely, and no severe discomfort was reported. The yield of HAMs was comparable with that of other studies involving healthy volunteers^5^ and was acceptable for ex vivo studies.^6^ The facility fee of the procedure ($1,598 from Medicare) was eliminated, making this a very cost-effective protocol.

Our protocol was designed to anesthetize the airway comprehensively with sequential lidocaine via nebulizer, atomizer, lollipop, and bronchoscope while ensuring a less than toxic lidocaine limit of 5 to 7 mg/kg.^7^ Most participants in our study achieved adequate anesthesia and did not experience coughing. However, the two self-limiting desaturation events were associated with coughing, suggesting suboptimal anesthesia in those patients. Although supplemental oxygen is routine in most bronchoscopy protocols, the omission of supplemental oxygen did not result in serious adverse events. Previous studies with patients with asthma reported no adverse events with higher doses of lidocaine (up to 8.2 mg/kg). Given that our study used lidocaine doses well below this threshold, future investigations could consider additional lidocaine to the turbinates and the use of oropharyngeal 10% lidocaine spray, which may be more effective than nebulized lidocaine.^8^^,^^9^ The optimal topical anesthesia protocol remains unknown and requires further study with comparison of delivery mechanisms. Future studies also may consider using thinner bronchoscopes to maximize patient comfort.

Our study has several limitations. First, we did not assess the adequacy of topical anesthesia before advancing the bronchoscope, which might have mitigated the single episode of moderate discomfort. Second, we did not evaluate operator comfort and satisfaction, which may be affected adversely compared with sedation-based approaches. Third, this study did not investigate patients with pulmonary disease, which will require further studies with more intensive intraprocedural monitoring. Finally, the nature of minor cell populations obtained was not investigated fully in this study and will be characterized further in subsequent studies.

In conclusion, this pilot study supports the feasibility and further investigation of research bronchoscopy without sedation. By minimizing the need for sedation, this approach has the potential to improve patient safety, to enhance procedural tolerability, and to facilitate research bronchoscopy.

## Funding/Support

Funding provided by the 10.13039/100000865Bill & Melinda Gates Foundation (OPP1130674) and the 10.13039/100000002National Institutes of Health [Grants NIA P01 AG051428 and NIAID 1R01AI136831-01A1].

## Financial/Nonfinancial Disclosures

None declared.

## References

[1] Du Rand I.A., Blaikley J., Booton R. (2013). British Thoracic Society guideline for diagnostic flexible bronchoscopy in adults: accredited by NICE. Thorax.

[2] Wahidi M.M., Jain P., Jantz M. (2011). American College of Chest Physicians consensus statement on the use of topical anesthesia, analgesia, and sedation during flexible bronchoscopy in adult patients. Chest.

[3] Matot I., Kramer M.R. (2000). Sedation in outpatient bronchoscopy. Respir Med.

[4] Morris L.G., Zeitler D.M., Amin M.R. (2007). Unsedated flexible fiberoptic bronchoscopy in the resident clinic: technique and patient satisfaction. Laryngoscope.

[5] Heron M., Grutters J.C., ten Dam-Molenkamp K.M. (2012). Bronchoalveolar lavage cell pattern from healthy human lung. Clin Exp Immunol.

[6] Pahari S., Arnett E., Simper J. (2023). A new tractable method for generating human alveolar macrophage-like cells in vitro to study lung inflammatory processes and diseases. mBio.

[7] Milman N., Laub M., Munch E.P. (1998). Serum concentrations of lignocaine and its metabolite monoethylglycinexylidide during fibre-optic bronchoscopy in local anaesthesia. Respir Med.

[8] Dhooria S., Chaudhary S., Ram B. (2020). A randomized trial of nebulized lignocaine, lignocaine spray, or their combination for topical anesthesia during diagnostic flexible bronchoscopy. Chest.

[9] Madan K., Biswal S.K., Tiwari P. (2019). Nebulized lignocaine for topical anaesthesia in no-sedation bronchoscopy (NEBULA): a randomized, double blind, placebo-controlled trial. Lung India.

